# Spatiotemporal Heterogeneity in Human *Schistosoma japonicum* Infection at Village Level in Hubei Province, China

**DOI:** 10.3390/ijerph16122198

**Published:** 2019-06-21

**Authors:** Juan Qiu, Rendong Li, Ying Xiao, Jing Xia, Hong Zhu, Yingnan Niu, Duan Huang, Qihui Shao, Ying Cui, Yong Wang

**Affiliations:** 1 Key Laboratory of Monitoring and Estimate for Environment and Disaster of Hubei Province, Institute of Geodesy and Geophysics, Chinese Academy of Sciences, Wuhan 430077, China; qiujuan@asch.whigg.ac.cn (J.Q.); huangduan@asch.whigg.ac.cn (D.H.); shaoqihui18@mails.ucas.ac.cn (Q.S.); cuiying17@mails.ucas.ac.cn (Y.C.); 2 Hubei Center for Disease Control and Prevention, Hubei Provincial Academy of Preventive Medicine, Wuhan 430079, China; xiao111ying@126.com (Y.X.); xiaj0608@163.com (J.X.) whzh2005@aliyun.com (H.Z.); 3 College of Earth and Planetary Sciences, University of Chinese Academy of Sciences, Beijing 100049, China; niuyingnan16@mails.ucas.ac.cn; 4 State Key Laboratory of Resources and Environmental Information Systems, Institute of Geographical Sciences and Natural Resources Research, Chinese Academy of Sciences, Beijing 100101, China

**Keywords:** *Schistosoma japonicum*, spatiotemporal heterogeneity, microscale, spatial analysis, multilevel growth model

## Abstract

The spatiotemporal dynamics of *Schistosoma japonicum*, combined with temporal heterogeneity among regions of different epidemic areal-types from a microscale viewpoint might capture the local change dynamics and thus aid in optimizing the combinations of precise schistosomiasis control measures. The prevalence data on schistosomiasis infection from 2007 to 2012 in the 30 most endemic counties of Hubei Province, Central China, were appended to the village-level administrative division polygon layer. Anselin local Moran’s I, a retrospective space–time scan statistic and a multilevel-growth model analysis framework, was used to investigate the spatiotemporal pattern of schistosomiasis resident infection rate (RIR) at the village level and how natural geographical environment influence the schistosomiasis RIR over time. Two spatiotemporal high-risk clusters and continuous high-rate clusters were identified mainly in the embankment region across flooding areas of lakes connected with the Yangze and Hanjiang Rivers. Moreover, 12 other clusters and outlier evolution modes were detected to be scattered across the continuous high-rate clusters. Villages located in embankment region had the highest initial values and most rapidly reduced RIRs over time, followed by villages located in marshland-and-lake regions and finally by villages located in hilly region. Moreover, initial RIR values and rates of change did significantly vary (*p* < 0.001 and *p* < 0.001, respectively) irrespective of their epidemic areal-type. These local spatiotemporal heterogeneities could contribute to the formulation of distinct control strategies based on local transmission dynamics and be applied in other endemic areas of schistosomiasis.

## 1. Introduction

*Schistosomiasis japonica* is caused by *Schistosoma japonicum*, one of the five trematode species [1] responsible for human and animal infections. It has considerable public health and economic significance in the People’s Republic of China [2,3]. Knowledge of the spatiotemporal heterogeneity of *Schistosomiasis japonica* on a small scale can provide important insights into local change dynamics and can thus aid in planning schistosomiasis control and elimination efforts. With the development of national and local surveillance systems, long-term temporal and micro-geographic level parasitological data are available which enable the characterization of changing patterns of disease risk in both temporal and spatial dimensions [4]. Different spatial analytical approaches can be used, including global Moran’s I and local Moran’s I statistics [5,6,7,8,9], spatial or space–time scan statistics [6,7,8,10,11,12], and the standard deviational ellipse [9,11]. Almost all studies on spatiotemporal patterns of schistosomiasis japonica focus on the county level because of the lack of village and township based vector data, and few studies have focused on the micro-scale level, especially the village-level [6]. The precision of spatial analyses is usually determined by the resolution of the underlying data (e.g., pixel size, time interval), and data at a coarse spatial resolution would fail to capture local heterogeneity [13].

The *Schistosomiasis japonica* endemic areas in China are divided into three types, based on the geographical characteristics of schistosomiasis endemic areas and the habitat environment of *Oncomelania hupensis*, the sole intermediate host of *S. japonicum*: (1) plain regions with waterway networks, (2) hilly and mountainous regions, and (3) marshland-and-lake regions and many subtypes such as embankment regions, hilly regions [14]. The comprehensive control strategy and prioritization of interventions should be varied between different endemic areas [15,16,17]. However, little is known about the temporal heterogeneity of *Schistosomiasis japonica* in different epidemic areal-types.

This study aimed to investigate the spatiotemporal patterns of human *S. japonicum* infection in most endemic counties in the Hubei Province of China at the village level. Anselin local Moran’s I statistics and a retrospective space–time scan statistic were used to determine the spatial distribution of *S. japonicum* infection over time. A multilevel-growth model was further used to explore the relationship between *S. japonicum* and epidemic areal-types and time factors.

## 2. Materials and Methods

### 2.1. Study Area

**Figure 1:**
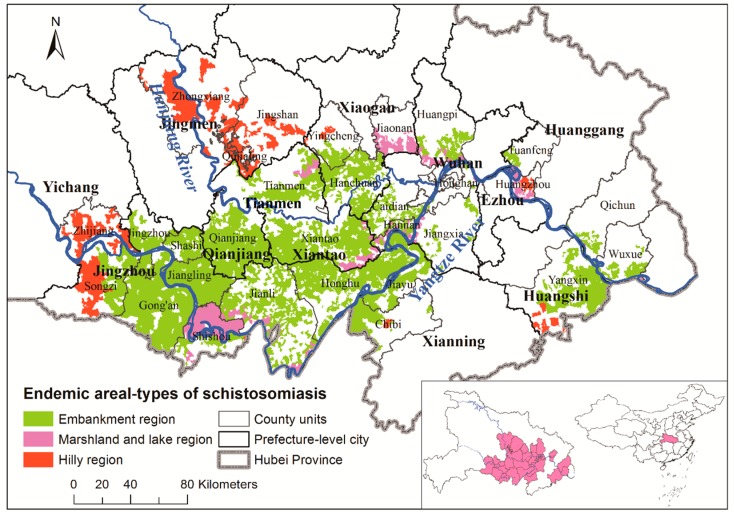
Study area and epidemic areal-types of schistosomiasis. The inset shows the location of the 30 most endemic counties in Hubei and in China. The geographical layers of Yangtze and Han Rivers are overlaid.

Hubei Province in Central China and the middle reaches of the Yangtze River (Figure 1) have included some of the most serious schistosomiasis outbreak regions of all time [12], and they had the highest estimated incidence of schistosomiasis in China up to 2014 [18]. Major rivers include the Yangtze River in the south and the Han River in the north. Though plains dominate half of Jianghan Province, as well as Southcentral Hubei, a series of mountains and hills dominate Western, Northeastern, and Southeastern Hubei. This study was carried out in the 30 most endemic counties with epidemic areal-types, including embankment, marshland-and-lake, and hilly regions in Hubei Province (Figure 1) [18,19].

### 2.2. Schistosomiasis Information and Geospatial Processing

The prevalence data on schistosomiasis infections in the 30 most endemic counties were collected from repeated cross-sectional surveys conducted annually between 2007 and 2012 by health professionals from the Hubei Institute of Schistosomiasis Control. This study targeted residents of each village who were 6–65 years old, and over 90% of these residents were screened every September to November using the indirect hemagglutination assay. Stool samples were subsequently collected from over 90% of the individuals with positive serological results, and used to perform the miracidium-hatching test [12]. Resident infection rates (RIRs) were calculated by multiplying the positive rate of serological tests with the positive rate of fecal tests. A total of 4682, 4689, 4710, 4722, 4714, and 4945 epidemic villages were surveyed from 2007 to 2012 in the 30 counties, respectively, and the coverage rate of epidemic villages is about 37%; all of them were appended to the village-level administrative division polygon layer based on the names of county, town, and village. An actual geospatial position in the administrative village layer was used to represent the “village” location through field investigation or by consulting local anti-schistosomiasis employers if a name matching the village-level administrative division was unavailable, for example because of a changed name, or because of not being an administrative village unit.

### 2.3. Spatial Statistical Approach

We performed cluster and outlier analyses (Anselin local Moran’s I) and a retrospective space–time scan statistic to investigate the spatiotemporal pattern of schistosomiasis RIR at village level.

Anselin local Moran’s I was used to identify spatial clusters and spatial outliers of regions with high or low RIR values by calculating a local Moran’s I value, a z-score, a *p*-value, and a code representing the cluster/outlier type for each region [20]. The z-scores and *p*-values represent the statistical significance of the computed index values using a Monte Carlo permutation approach [21,22]. Five cluster/outlier types for local autocorrelation exist: (1) HH (high cluster) indicates that a village and its neighbors had high RIR; (2) LL (low cluster) indicates that a village and its neighbors had low RIR; (3) HL indicates that a village was a high outlier among villages with low RIR; (4) LH indicates that a village was a low outlier among villages with high RIR; and (5) ‘not significant’ indicates no spatial autocorrelation. To determine how spatial autocorrelation may evolve with time and location, cluster and outlier analysis results from 2007 to 2012 were joined by a ‘-’, and then all combined types were classified as “HH,” “HL,”“LH,” “LL,” “H to L,” “L to H,” and “Others” (detailed classification in Supplemental Table S1). For instance, “HH-HH-HH-HH-HH-HL” means HH type from 2007 to 2011 and HL type in 2012 and is classified as “H to L.”

The space–time scan statistic using a moving cylindrical window with circular (or elliptic) geographic base and with height corresponding to time is widely used in disease studies to identify statistically significant high-risk spatiotemporal clusters [8,11,23,24]. SaTScan™ (version 9.4, M. Kulldorff, Harvard Medical School, Boston and Information Management Services Inc., Silver Spring, MD, USA.) was used to identify high-risk clusters defined by circular shapes, constrained to clusters with both RIR values and study periods less than 50% [10]. The RIRs showed skewed distributions and were thus normalized by log (base 10) transformation after adding the mean RIR limit to allow for zeros. The spatiotemporal distribution of log transformed RIR was assessed using the normal model designed for continuous data [25]. Monte Carlo simulations (999 times) were used for significance testing at the 0.05 level. The attributed values for these output circles included X and Y coordinates for the centers and cluster radius.

All spatial processing and mapping were carried out in ArcGIS 10.1 (ESRI Inc., Redlands, CA, USA).

### 2.4. Multilevel-Growth Model

To evaluate the impact of the natural geographical environment (epidemic areal-type) on differences in schistosomiasis RIR over time, a multilevel-growth model analysis framework was developed with “occasions of measures” assigned to level 1 and “village data” to level 2 [26,27,28]. We first coded the predictors to aid substantive interpretations as follows: time (rectilinear: Year = 0, 1…, 6 representing 2007, 2008, …, 2012, respectively) and epidemic areal-type (0—Embankment region, 1—Marshland-and-lake region, 2—Hilly region). A taxonomy of multilevel-growth models was specified following methods described by Singer and Willett (2003) [28].

The unconditional means model, also referred to as an intercept-only model or a null model [29], is expressed as:

(1) $${\mathrm{RIR}}_{ij}=\gamma_{00}+\left({𝒰}_{0j}+e_{ij}\right)$$

where ${\mathrm{RIR}}_{ij}$ represent the village *j*’s schistosomiasis RIR on occasion *i*, ${𝒰}_{0j}$ and $e_{ij}$ represent the village- and occasion-specific random effects, respectively. These random effects were assumed to have a normal distribution.

The unconditional growth model, including predictor ${\mathrm{Year}}_{ij}$ into the unconditional means model, is expressed as:

(2) $${\mathrm{RIR}}_{ij}=\gamma_{00}+\gamma_{10}{\mathrm{Year}}_{ij}+\left({𝒰}_{0j}+{𝒰}_{1j}{\mathrm{Year}}_{ij}+e_{ij}\right)$$

Adding a village-level covariate, $\mathrm{Areal}\_{\mathrm{type}}_{j}$, indicating the schistosomiasis epidemic areal-type of village *j*, leads to:

(3) $${\mathrm{RIR}}_{ij}=\gamma_{00}+\gamma_{10}{\mathrm{Year}}_{ij}+\gamma_{01}\mathrm{Areal}\_{\mathrm{type}}_{j}+\left({𝒰}_{0j}+{𝒰}_{1j}{\mathrm{year}}_{ij}+e_{ij}\right)$$

Cross-level interactions can be assessed as:

(4) $${\mathrm{RIR}}_{ij}=\gamma_{00}+\gamma_{10}{\mathrm{year}}_{ij}+\gamma_{01}\mathrm{Areal}\_{\mathrm{type}}_{j}+\gamma_{11}{\mathrm{year}}_{ij}*\mathrm{Areal}\_{\mathrm{type}}_{ij}\;+\left({𝒰}_{0j}+{𝒰}_{1j}{\mathrm{year}}_{ij}+e_{ij}\right)$$

where ${𝒰}_{0j}$ and ${𝒰}_{1j}$ are bivariate normally distributed with a mean of 0 and constant variance.

All analyses were conducted using SAS (PROC MIXED) version 9.3 (SAS Institute, Inc., Cary, NC, USA).

## 3. Results

### 3.1. Spatiotemporal Pattern Analysis

**Figure 2:**
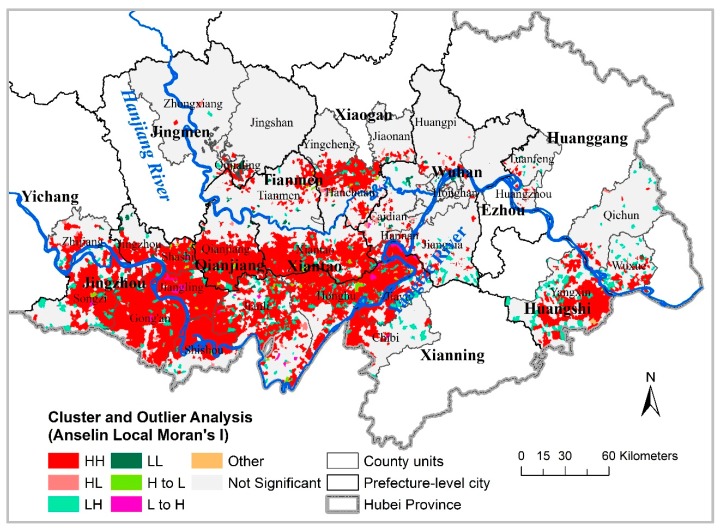
Cluster and outlier evolution for schistosomiasis RIR (resident infection rate) from 2007 to 2012 in the 30 most endemic counties, Hubei Province.

Figure 2 depicts the heterogeneous geospatial distribution of RIR clusters and outliers identified by local Moran’s I over time, corresponding mainly to villages with high-rate clusters (HH; red) shown in the descriptive maps. Specifically, major high-risk clusters occurring between 2007 and 2012 (HH) comprised 3068 villages (Table 1), accounting for 77% of the total number of significant (0.05 level) villages and included a large geographic area in Southcentral Hubei Province (i.e., Jingzhou, Qianjiang, and Xiantao cities; Figure 2) and two small areas in Hanchuan and Yangxin. A few sporadic villages among the HH villages were identified as LH. Two others unchanged types (HL and LL) throughout the seven years ware mainly located in Caidian and Qujialing. In a small number of cases, comprising 93 villages (Table 1) scattered across Jiangzhou, Qianjiang, Xiantao, and Hanchuang (Figure 2), cluster/outlier types changed over time (L to H, and H to L).

**Figure 3:**
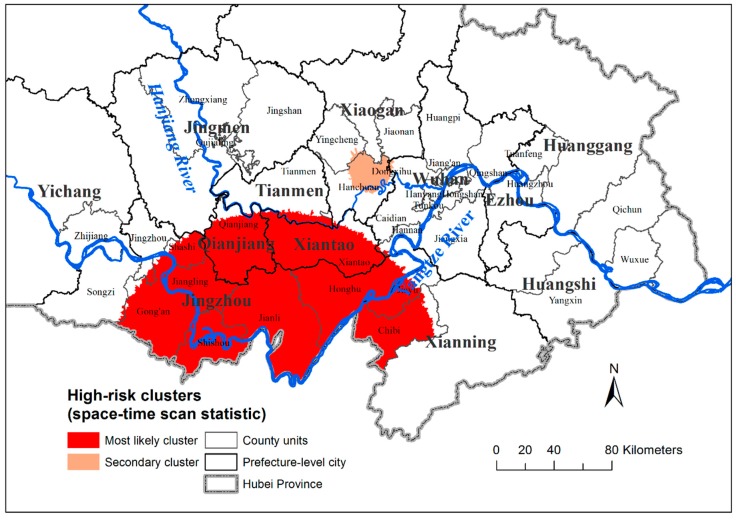
Mapping of significant spatiotemporal clusters of schistosomiasis RIR, by administrative village.

Space–time scan analysis identified two spatiotemporal high-risk clusters (Figure 3 and Table 2). Primary clusters were detected within the period 2007–2009 and represented 2184 villages distributed in 10 counties. These were mainly embankment region epidemic areal-types in the middle-south of Hubei Province. Secondary clusters of embankment region epidemic areal-types, representing 125 villages and also throughout 2007–2009, were located in Northeastern Hanchuan. Both clusters remained statistically significant (*p* < 0.001). The two spatiotemporal high-risk clusters were basically consistent with the high cluster (HH) of cluster and outlier analysis.

### 3.2. Multilevel-Growth Model

Overall, 4379 surveyed villages from 2007 to 2012 were eligible for model inclusion. A total of 3501 villages were located in embankment regions, 446 in hilly regions, and 432 in marshland-and-lake regions, respectively (Figure 1). Table 3 summarizes the results of multilevel-growth models.

#### 3.2.1. Unconditional Means Model

The variance components of the unconditional means model showed statistically significant variance associated with villages (${\hat{\sigma }}_{𝓊0}^{2}\;$= 1.045; *p* < 0.001) and statistically significant residual variance (${\hat{\sigma }}^{2}\;$= 1.275; *p* < 0.001). The intraclass correlation coefficient calculated as $\frac{1.045}{1.045+1.2757}=0.45$ indicated that approximately 45% of RIR variance arose from differences between villages [30,31].

#### 3.2.2. Unconditional Growth Model

The unconditional growth model resulted in significant fit improvement with reduced −2LL, AIC, and BIC compared with the empty model (Table 3). The linear fixed effect of time differed significantly and negatively from zero (${\hat{\gamma }}_{10}\;$= −0.351; *p* < 0.001), indicating that villages’ RIR values declined by about 0.35% per year on average, irrespective of their epidemic areal-type. Adding the fixed and random effects for time accounted for 73.6% of the explainable variance, as calculated using the Raudenbush and Bryk method [32].

#### 3.2.3. Final Model

Table 3 summarizes the goodness-of-fit of the RIR models. The −2LL, AIC, and BIC for models, including the cross-level interactions model, was smaller than the corresponding value obtained for the other models, indicating that the assessing cross-level interaction model fitted the data better than the other models. After exploring the possibility that time (year), areal-type, and their interactions predicted differences in RIR values, the variance of mean initial status (${\hat{\sigma }}_{𝓊0}^{2}\;$= 3.809; *p* < 0.001) revealed that villages significantly varied in their initial RIR values and that the variance of rate of change (${\hat{\sigma }}_{𝓊1}^{2}\;$= 0.136; *p* < 0.001) indicated that village rates of change varied significantly. The estimated covariance (${\hat{\sigma }}_{𝓊01}^{2}\;$= −0.716; *p* < 0.001) was negative, indicating that villages with low initial RIR values tended to have higher rates of change, with statistical significance. The mean initial RIR value (${\hat{\gamma }}_{00}$) was 3.418%. A significant negative effect of areal-type on initial status (${\hat{\gamma }}_{01}\;$= −0.896; *p* < 0.001) showed that these initial RIR values differed among the three epidemic areal-types, with higher initial values in embankment regions, medium initial values in marshland-and-lake regions, and lower initial values in hilly regions. The effect of areal-type on rate of change (${\hat{\gamma }}_{11}\;$= 0.143; *p* < 0.001) was positive, indicating that RIR growth rate among epidemic areal-types significantly differed, on average, by 0.143% per year. Specifically, villages located in embankment regions reduced their RIR most rapidly over time, followed by villages located in marshland-and-lake regions and then by villages located in hilly regions.

## 4. Discussion

The heterogeneous geographic pattern of schistosomiasis RIR was found based on this spatial statistical approach, including HH clusters detected by local Moran’s I statistics and primary clusters detected by the space–time scan statistics. High-risk clusters for the schistosomiasis RIR were mainly identified in flooding areas of lakes connected to the Yangze and Hanjiang Rivers, particularly in the cities of Jingzhou, Qianjiang, and Xiantao, and in the embankment regions, which were consistent with the cluster regions at county level [12] and provided more details. These areas have natural geographical conditions and socioeconomic factors favorable to schistosomiasis; i.e., an eco-hydrological environment suitable for the survival of the intermediate snail host and its multiplication [33,34]. The plain region with waterway networks evolved from a typical lake and marshland to a schistosomiasis epidemic region connected by rivers and lakes because of farmland reclamation from lakes and large-scale excavation channels after the mid-1970s. Accordingly, the snail distribution evolved linearly from sheet distributions in the bottomland along the river and lakes, and along the ditches, irrigation canals, and rivers with conducive humidity, vegetation, elevation and flow velocity. The broad dispersion of snails, and the frequent flooding, as well as people’s frequent contact with contaminated water due to traditional livelihoods and lifestyles, is a challenge to effective control [35].

The results of the cluster and outlier evolution analyses for the 2007–2012 schistosomiasis RIR had 8 combination modes (including “not significant”) and various spatial distributions of combination modes (Figure 2 and Table 1), indicating that the different risk patterns require distinct control strategies and decision-making processes. This finding may be due to the different infectious sources and populations of schistosomiasis infection in different endemic areas and periods [36]. Additionally worth taking into account, aside from HH clusters and primary clusters, were secondary clusters located in Northeast Hanchuan (Figure 3), and villages with initially low rates that became high rates within a year (L to H, Figure 2), together with adjacent villages. 

After exploring the effect of time (year), areal-type, and their interactions on RIR values, village RIR values significantly declined by about 0.537% per year on average (Table 3), which provided some explanation for the periods of two spatiotemporal high-risk clusters (Figure 3 and Table 2) detected by space–time scan analysis was the first three years. We also found temporal heterogeneity of schistosomiasis RIRs between the three epidemic areal-types. The embankment region with the widest area had the highest RIR values in the first year, which was possibly due to more frequent human activity and repeated infections compared to the marshland-and-lake, and hilly regions. Meanwhile, villages located in embankment regions most rapidly reduced their RIR over time, which may be because more attention was paid to integrated control strategies [37]. Significant differences existed in the initial RIR values and rates of change between the three epidemic areal-types, as well as within villages located in the same epidemic areal-type, which reminds us again that suitable comprehensive control measures must take account of the local epidemic situation rather than depend upon a uniform strategy.

In addition, villages in all epidemic regions with higher initial RIR values and faster rates of decline may, with time, have a sustained low prevalence of schistosomiasis. A low transmission of schistosomiasis has been detected in the hilly and mountainous endemic regions of Sichuan [38]. Consequently, new challenges toward the elimination of schistosomiasis japonica transmission in China may ensue because of the low sensitivity/specificity of current diagnostic tools for infections, praziquantel resistance, and climate change [38,39,40,41].

These analyses require more updated data for confirmation, which are not available, which is one limitation of our study. Moreover, two spatial analysis techniques were used to investigate spatial patterns, and although both results are broadly in agreement, no comparative study was conducted among different statistical methods because of their large diversity and different focuses on spatial patterns.

China continues to invest substantially in control activities aimed at eliminating schistosomiasis by 2020 [42]. Currently, the elimination of schistosomiasis depends on the sustained implementation of an integrated control strategy with great emphasis on the control of infectious sources [39,43,44]. For example, aspects to avoid infection in humans include: Improving sanitation by supplying tap water and building lavatories and latrines, providing boats with fecal-matter containers, and implementing an intensive health-education program and vaccination for long-term prevention [45]. Given all these different potential strategies, the importance of suiting the precise targeted control and priority of interventions to the local conditions is of concern [15,16,17].

## 5. Conclusions

This study systematically and exhaustively investigated the spatiotemporal dynamics of *Schistosoma japonicum* and temporal heterogeneity among regions of different epidemic areal-types by using spatial statistical analysis and multilevel-growth models at the microscale. Its high spatial resolution captured local heterogeneity, which could aid in regional targeting of schistosomiasis control measures and the spatiotemporal analysis method could be applied in other endemic areas of schistosomiasis.

## Figures and Tables

**Table 1:** Combined cluster/outlier found by the local Moran’s I analysis for schistosomiasis RIR (resident infection rate).

Type	No. of Villages	Description of Types
HH	3068	a high cluster that is statistically significant (0.05 level) in all years or in some years from 2007 to 2012
LH	631	a low outlier that is statistically significant (0.05 level) in all years or in some years from 2007 to 2012
HL	90	a high outlier that is statistically significant (0.05 level) in some years from 2007 to 2012
LL	97	a low cluster that is statistically significant (0.05 level) in some years from 2007 to 2012
H to L	52	a high-value village or its neighbors converted into a low-value village or its neighbors, i.e., a high cluster converted into a high outlier (HH to HL), or a high cluster converted into a low outlier (HH to LH), or a low outlier converted into a low cluster (LH to LL)
L to H	41	a low-value village or its neighbors converted into a high value village or its neighbors, i.e., a high outlier converted into a high cluster (HL to HH), or a low outlier converted into a high cluster (LH to HH)
Others	8	Others (Table S1)

**Table 2:** Significant spatiotemporal clusters of schistosomiasis RIR as defined by space–time scan statistic in the 30 most endemic counties, Hubei Province, 2007–2012.

Cluster ^1^	Period	Cluster Location	Cluster Radius (km)	No. Villages	County	LLR ^2^	*p* Value
1	2007–2009	29.485 N, 112.956 E	113.94	2184	Qianjian, Xiantao, Honghu, Chibi, Jianli, Shishou, Gong’an, Jiangling, Shashi, Jiayu	3764.32	<0.001
2	2007–2009	30.720 N, 113.766 E	13.72	125	Hanchuan	176.65	<0.001

^1^ The most likely or primary clusters (1) and secondary clusters (2) were detected by the LLR. The most likely cluster was defined as the one with the maximum LLR. ^2^ LLR: loglikelihood ratio test.

**Table 3:** Results of fitting multilevel-growth models to schistosomiasis RIR in 4379 villages in the 30 most endemic counties, Hubei Province, China.

	Unconditional Means (Empty Model)	Unconditional Growth	Adding a Village-Level Covariate	Assessing Cross-Level Interactions
**Fixed Effects**				
Initial Status (${\hat{\gamma }}_{00}$)	1.374	2.252	2.505	3.418
Year (Rate of Change) (${\hat{\gamma }}_{10}$)		−0.351	−0.351	−0.537
Areal-type (Effect of areal-type on initial status) (${\hat{\gamma }}_{01}$)			−0.194	−0.896
Areal-type (Effect of areal-type on rate of Change) (${\hat{\gamma }}_{11}$)				0.143
**Random Effects**				
Level 1 (within village)				
Residual (${\hat{\sigma }}^{2}$)	1.275	0.337	0.337	0.337
Level 2 (between village)				
Village Mean Initial Status (${\hat{\sigma }}_{𝓊0}^{2}$)	1.045	4.142	4.013	3.809
Village Mean Rate of Change (${\hat{\sigma }}_{𝓊1}^{2}$)		0.145	0.145	0.136
Rate of change covariance (${\hat{\sigma }}_{𝓊01}^{2}$)		−0.769	−0.758	−0.716
**Fit Statistics**				
−2LL	88,737.3	61,858.3	61,586.1	61,354.3
AIC	88,743.3	61,870.3	61,600.1	61,370.3
BIC	88,762.4	61,908.6	61,644.8	61,421.4

## Supplementary Materials

The following are available online at https://www.mdpi.com/1660-4601/16/12/2198/s1, Table S1: Combined clusters and outliers evolution modes and their categories.
