# Supplementary material for: Spatiotemporal Heterogeneity in Human Schistosoma japonicum Infection at Village Level in Hubei Province, China
Source: Int J Environ Res Public Health. 2019 Jun 21;16(12):2198. doi: 10.3390/ijerph16122198 (PMC6617067; doi:10.3390/ijerph16122198)
Supplement: Supplementary file 1 [file ijerph-16-02198-s001.pdf]

Table S1. Combined clusters and outliers evolution modes and their categories. Cluster and outlier analysis results from 2007 to 2012 were joined by a semicolon and then been classified. For instance, "HH-HH-HH-HH-HH-HL" means HH types from 2007 to 2011 and HL type in 2012, and classified as "H to L".

Category/Combined cluster or outlier type	No. of villages
**HH**	**3068**
HH-HH-HH-HH-HH-HH	935
HH-----	52
-HH----	112
--HH---	17
----HH-	13
-----HH	114
HH-HH----	454
HH--HH---	1
HH-----HH	8
-HH-HH---	2
-HH----HH	10
--HH-HH--	14
--HH--HH-	1
--HH---HH	5
---HH-HH-	2
---HH--HH	3
----HH-HH	64
HH-HH-HH---	12
HH-HH--HH--	1
HH-HH---HH-	10
HH-HH----HH	68
HH--HH-HH--	2
HH----HH-HH	9
-HH-HH-HH--	1
-HH-HH---HH	1
-HH---HH-HH	8
--HH-HH-HH-	20
--HH-HH--HH	11
--HH--HH-HH	15
---HH-HH-HH	43
HH-HH-HH-HH--	9
HH-HH-HH--HH-	5
HH-HH-HH---HH	3
HH-HH--HH--HH	2
HH-HH---HH-HH	40
HH--HH-HH-HH-	5
HH--HH-HH--HH	2
HH--HH--HH-HH	2
HH---HH-HH-HH	6
-HH-HH-HH-HH-	5
-HH-HH-HH--HH	1
-HH-HH--HH-HH	2
-HH--HH-HH-HH	2
--HH-HH-HH-HH	683
HH-HH-HH-HH-HH-	25
HH-HH-HH-HH--HH	10
HH-HH-HH--HH-HH	17
HH-HH--HH-HH-HH	32
HH--HH-HH-HH-HH	89
-HH-HH-HH-HH-HH	120
**HL**	**90**
HL-----	1
-HL----	9
--HL---	6
---HL--	1
----HL-	10
-----HL	22
HL-HL----	9

-HL----HL	1
---HL-HL-	1
----HL-HL	11
--HL-HL-HL-	3
--HL-HL--HL	3
--HL--HL-HL	1
---HL-HL-HL	1
--HL-HL-HL-HL	10
HL-HL-HL--HL-HL	1
**LL**	**97**
-LL----	6
---LL--	2
-----LL	39
-LL----LL	1
---LL-LL-	2
--LL-LL-LL-	1
---LL-LL-LL	10
--LL-LL-LL-LL	36
**LH**	**631**
LH-LH-LH-LH-LH-LH	21
LH-----	36
-LH----	71
--LH---	4
---LH--	10
----LH-	9
-----LH	50
LH-LH----	128
LH--LH---	2
LH-----LH	1
-LH----LH	1
--LH-LH--	21
--LH--LH-	2
--LH---LH	9
---LH-LH-	3
---LH--LH	6
----LH-LH	20
LH-LH----LH	2
LH----LH-LH	1
--LH-LH-LH-	24
--LH-LH--LH	25
--LH--LH-LH	2
---LH-LH-LH	9
LH-LH-LH-LH--	3
LH-LH--LH-LH-	1
LH-LH---LH-LH	1
LH--LH-LH-LH-	1
LH---LH-LH-LH	1
-LH-LH-LH-LH-	2
--LH-LH-LH-LH	153
LH-LH-LH-LH-LH-	2
LH-LH-LH-LH--LH	3
LH-LH--LH-LH-LH	2
LH--LH-LH-LH-LH	1
-LH-LH-LH-LH-LH	4
**H to L**	**52**
**H to L (HH to LH)**	
HH-HH-HH-HH-HH-LH	1
HH-HH-HH-HH--LH	1
HH--HH-HH-HH-LH	1
--HH-HH-HH-LH	1
HH-HH-HH-LH-LH-LH	1
HH-HH--LH--	1
HH-HH----LH	4
--HH--HH-LH	1

---HH-HH-LH	1
HH-HH-LH-LH--	2
HH-HH-LH-LH-LH-	2
HH-HH-LH-LH--LH	1
HH-HH--LH-LH-LH	1
HH-HH-LH-LH-LH-LH	7
-HH----HL	2
HH--LH-LH--	1
-HH--LH--LH	1
-HH--LH-LH-LH	1
--HH-LH-LH-LH	2
HH--LH-LH-LH-LH	1
-HH-LH-LH-LH-LH	3
**H to L (HH to HL)**	
HH-HH---HL-	2
HH-HH----HL	1
--HH-HH-HL-	1
HH-HH-HL-HL--	2
HH-HH---HL-HL	1
HH-HH-HL-HL-HL-	1
HH-HH-HL-HL-HL-HL	3
-HH---HL-HL	1
-HH-HL-HL--HL	1
HH--HL-HL-HL-HL	1
-HH-HL-HL-HL-HL	1
**H to L (LH to LL)**	
-LH-LL-LL--LL	1
**L to H**	**41**
**L to H (HL to HH)**	
-HL----HH	1
HL--HH--HH-HH	1
HL--HH-HH-HH-HH	1
-HL-HH-HH-HH-HH	3
HL-HL-HH---	1
HL-HL----HH	2
HL-HL-HH-HH-HH-HH	5
**L to H (LH to HH)**	
LH-HH----	2
-LH----HH	1
-LH-HH--HH-	1
LH--HH-HH-HH-HH	6
-LH-HH-HH-HH-HH	6
--LH-LH-HH-HH	1
LH-LH-HH-HH-HH-	1
LH-LH-HH-HH-HH-HH	9
**Others**	**8**
HH-HH-HH-HH-LH-HH	2
HH-HH-HH-LH-HH-HH	1
HH-HH-LH---HH	1
HH--HH-LH-HH-HH	1
HH-HL-HH-HH-HH-HH	1
HH-LH-HH-HH-HH-HH	2
**Sum**	**3987**
